# A century of exercise physiology: key concepts in muscle energetics

**DOI:** 10.1007/s00421-022-05070-7

**Published:** 2022-10-22

**Authors:** C. J. Barclay

**Affiliations:** School of Biomedical Sciences, University of Queensland, St Lucia, Brisbane, Australia

**Keywords:** Skeletal muscle energetics, Muscle heat production, Muscle physiology, Muscle contraction

## Abstract

In the mid-nineteenth century, the concept of muscle behaving like a stretched spring was developed. This elastic model of contraction predicted that the energy available to perform work was established at the start of a contraction. Despite several studies showing evidence inconsistent with the elastic model, it persisted into the twentieth century. In 1923, W. O. Fenn published a paper in which he presented evidence that appeared to clearly refute the elastic model. Fenn showed that when a muscle performs work it produces more heat than when contracting isometrically. He proposed that energy for performing work was only made available in a muscle as and when that work was performed. However, his ideas were not adopted and it was only after 15 years of technical developments that in 1938 A. V. Hill performed experiments that conclusively disproved the elastic model and supported Fenn’s conclusions. Hill showed that the rate of heat production increased as a muscle made the transition from isometric to working contraction. Understanding the basis of the phenomenon observed by Fenn and Hill required another 40 years in which the processes that generate force and work in muscle and the associated scheme of biochemical reactions were established. Demonstration of the biochemical equivalent of Hill’s observations—changes in rate of ATP splitting when performing work—in 1999 was possible through further technical advances. The concept that the energy, from ATP splitting, required to perform work is dynamically modulated in accord with the loads a muscle encounters when contracting is key to understanding muscle energetics.

## Introduction

The key concept in muscle energetics that is examined in this article is that muscle energy expenditure is determined by the mechanical loading a muscle encounters as it contracts. From the mid-nineteenth century until the 1930s, a contrasting idea held sway. Then it was thought that muscle energy expenditure was determined simply by the length of a muscle at the start of a contraction and that the mechanical behaviour of active muscle, and its associated energetics, could be understood by considering the muscle to act like a stretched spring. This was termed the elastic theory of contraction. Within that period, in 1923, a 30-year old American, Wallace O. Fenn, on a travelling fellowship to the UK wrote a paper in which he presented evidence that vigorously challenged the elastic theory. Fenn’s work might have been expected to overturn the elastic theories but that was not the case. Instead, the elastic theory continued to be used and elaborated. In this article, Fenn’s work is described in the context of the foundational work of European scientists in the nineteenth century and the prolonged pursuit of conclusive evidence about the validity or otherwise of the elastic theory by A. V. Hill in the twentieth century.

Central to this field of research was the use of measurements of the heat produced by muscles when they contract to examine fundamental aspects of muscle contraction. The application of physics to the study of biology in the nineteenth century, in particular knowledge of the behaviour of elastic materials and developing ideas about conservation of energy, provided both the impetus to explore muscle heat production in relation to activity and the development of a framework within which muscle heat output could be understood. The ultimate rejection of those ideas opened the field for a new framework to encompass the production of heat by active muscle and the processes underlying contraction.

There are recent comprehensive reviews covering biochemical aspects of energetics and the relationship between muscle heat production and biochemical changes (Barclay 2015a; Barclay and Loiselle 2021; Kushmerick 1983; Woledge et al. 1985) and on muscle biochemistry during exercise (e.g. Brooks 2012). It is not intended that this review overlap any more than necessary with such work. Instead, the current work will focus on the role that measurements of muscle heat output have played in the evolution of one of the key concepts in muscle energetics. Heat measurements are complicated and many of the technical minutiae that are often prominent in papers in the area are of somewhat esoteric interest. Consequently, it is not intended to focus on those aspects but rather to attempt to give a broad view of how, in this particular case, advances in physiological knowledge were slowed by the time taken to both overcome embedded ideas and to make necessary technical advances.

## Foundations of research on muscle energetics in the nineteenth century

### The elastic model of muscle mechanics

**Fig. 1:**
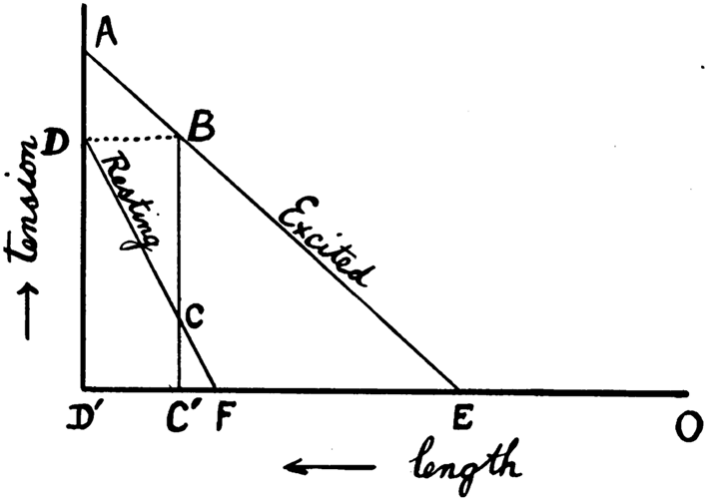
Hill’s depiction of the elastic model. Diagram shows the force-extension relationships for relaxed and stimulated muscle. x-axis: muscle length, increasing from right to left; y-axis, muscle force output (or tension). Line labelled relaxed: force-extension relation for relaxed muscle; line labelled “excited”, force-extension relationship for stimulated muscle. If a relaxed muscle at length C′, with elastic force C, is stimulated, it becomes a new elastic body with elastic force of B, giving a potential energy equal to the area enclosed by EC′B. If muscle length does not change during a contraction (i.e. an isometric contraction), all that energy will be converted into heat. If the muscle shortens, part or all of that energy will be converted into work and energy not converted into work will be converted into heat. Therefore, the greatest amount of heat will be produced in an isometric contraction. From Hill (1913a), used with permission from John Wiley and Sons

The modern approach to the study of muscle physiology arose in Europe in the 1830s and 1840s (for reviews of work in this and earlier eras, see Hill 1959, 1965b; Needham 1971). An important step was Theodor Scwhann’s 1835 demonstration that an isolated frog muscle produced its greatest force upon stimulation when close to its in vivo length and that less force was produced when the muscle was held at shorter lengths. That observation prompted Johannes Muller (Berlin, Germany) to propose that muscle be considered as an elastic body, with its force output reflecting its extension (or length). Eduard Weber (Liepzig, Germany) extended that idea by stating that the elastic properties of muscle altered upon stimulation so that the stimulated muscle behaved as a “new elastic body”, that is, with different elastic properties to an unstimulated muscle. Muller and Weber compared a stimulated muscle to a stretched spring and as such the muscle contained elastic potential energy (Fig. 1). If the muscle lifted a load, and in so doing shortened, then some of the elastic energy would be converted into mechanical work. Any elastic energy not converted into work would be dissipated as heat when the muscle force declined during relaxation. In this model, the only determinant of the amount of energy liberated as heat and work was the length of the muscle at the time it was stimulated. This simple phenomenological model of muscle mechanics, and variations of it, was to persist for almost 100 years.

#### The first measurements of isolated muscle heat output

The idea that the elastic energy would appear as either work or heat arises from the idea of conservation of energy which was a subject of much interest in European science at the time. Hermann Helmholtz (Berlin, Germany) had been seeking examples supporting the principle of energy conservation and one of these related to muscle. Increases in muscle temperature during activity had been recorded in humans and animals (Becquerel and Breschet 1835) and (Liebig 1842) proposed that the heat produced by muscle arose from chemical changes occurring within the muscle. Helmholtz recognized that the warming of muscles could occur by the transfer of heat to them from circulating blood. To address that possibility, he measured the contraction-induced change in temperature of a frog muscle preparation isolated from its blood supply and stimulated via its nerve (Helmholtz 1848). Helmholtz measured the temperature change using a thermopile and a galvanometer to measure the current flowing through the thermopile. The thermopile consisted of three pairs of thermocouples in series, with one of each pair (the “active” thermocouple) embedded in the stimulated muscle and the other (the “reference” thermocouple) embedded in the unstimulated contralateral muscle. This system could easily resolve the differences in temperature between the two muscles (up to 0.2 ºC) that occurred in the muscle in response to tetanic stimulation. Helmholtz’s method of using a thermopile to measure changes in muscle temperature, and thus to potentially calculate the amount of heat produced by the muscle, paved the way for subsequent workers to address the validity of the elastic model.

### Measurements of heat and work output to assess the elastic model

Within the framework of the elastic theory, it was thought that as force developed in an isometric contraction, heat was produced from the, presumably chemical, processes that accounted for the development of the elastic potential energy (Hill 1913a; Hill and Hartree 1920). If the muscle performed no work, as in an isometric contraction, then when force declined during relaxation an amount of heat equivalent to the elastic potential energy would be produced and the total heat output would be the sum of the heat from process creating the elastic energy and the heat from the dissipation of the elastic energy. If the muscle performed work during the contraction, some of the potential energy would be converted to work, and less heat would arise from the decay of elastic potential energy. Therefore, if the elastic theory was correct, less heat would be produced in a working contraction than in an isometric contraction but the total energy output (i.e. heat + work) would be the same. The predictions of the elastic model in term of heat output and total energy output are summarised in Table 1.

**Table 1:** Predictions of energy output from elastic model

	Heat output	Total energy output
Isometric	$Q_{D} + Q_{PE}$	$Q_{D} + Q_{PE}$
Working	$Q_{D} + (Q_{PE} - W)$	$$\begin{gathered} Q_{D} + (Q_{PE} - W) + W \hfill \\ = Q_{D} + Q_{PE} \hfill \\ \end{gathered}$$
$\Delta_{{{\text{Isom}} - {\text{Working}}}}$	W	0

*Q*_*D*_ heat produced in developing elastic potential energy; *Q*_*PE*_ thermal equivalent of elastic potential energy; *W* work performed; $\Delta_{{\text{Isom - Working}}}$, difference in heat or total energy output between working and isometric contractions. *Q* thermodynamic symbol for heat (Jensen 2010)

A substantial body of work was produced between the 1840s and 1910 by European scientists interested in heat production by animals in general and muscles in particular. This work was reviewed by Blix (1902). Three scientists in particular focussed on establishing whether less heat was produced in contractions in which work was performed than in an isometric contraction: Rudolph Heidenhain (Breslau, Germany), Adolph Fick (Wurzburg, Germany) and Magnus Blix (Lund, Sweden). Both Heidenhain (1869) and Fick (1892) found that *more* heat was produced in working contractions than in isometric contractions, at odds with the elastic model. Fick also proposed that during shortening a chemical process takes place, which gives off partly mechanical work and partly heat. Blix (1902) summarised Fick’s conclusion,

*… it is the working capacity of the muscle that demands the consumption of its chemical energy. The maintenance of tension without shortening or shortening without tension requires … less energy conversion than the production of mechanical work.*

Although Blix (1902) was able to replicate Fick’s results, he thought that Fick’s conclusion was wrong. Blix argued that Fick had not taken account of the effect of muscle length on heat output and that differences in length between the isometric and working contractions had led to the wrong conclusion. When Blix took muscle length differences into account, he concluded, “The isometric tetanus gives the greatest heat. It is the length of the muscle which is decisive for heat production.” (Blix 1902).

These early studies were confounded not only by effects of changes in muscle length but also by the many uncertainties that arose from developing new devices (thermopiles, galvanometers, stimulators and lever systems for loading muscles) (Bürker 1908) and a new preparation. There was considerable experimentation with different frog muscles, in vitro conditions to keep muscles alive, ways to minimise fatigue, characterising seasonal variations in frog muscle performance and dealing with non-uniform temperature changes along the length of muscles (Blix 1902; Bürker 1919). The European scientists had particularly focussed their technical developments on improving the sensitivity of temperature recordings: from Helmholtz in 1847 to Blix in 1902, the resolution had improved from ~ 10^–3^ ºC to 10^–6^ ºC (Bürker 1908). However, at least for some of the devices, speed of response was sacrificed for the high sensitivity. For instance, a thermopile/galvanometer system built by Blix and subsequently used by Hill, had a time constant of ~ 20 s (Hill 1910). Obtaining steady outputs from galvanometers, in particular, required considerable damping, and thus slowing, of their responses (Hill 1913b). The heat measuring devices were typically subject to considerable environmental disturbances, both electrical and thermal, prompting Nawalichin (1877), from Heidenhain’s laboratory, to say that,

*“It requires the coincidence of the most favourable conditions, often beyond the will of the experimenter, in order to arrive at flawless and trustworthy observations, and for me it requires an unreasonably long time to acquire the necessary certainty in the evaluation of what is seen”.*

These are sentiments with which many Physiologists will be familiar! In this case, it was already apparent that measuring muscle heat production was technically challenging; in that one respect, the method remains unchanged today.

**Fig. 2:**
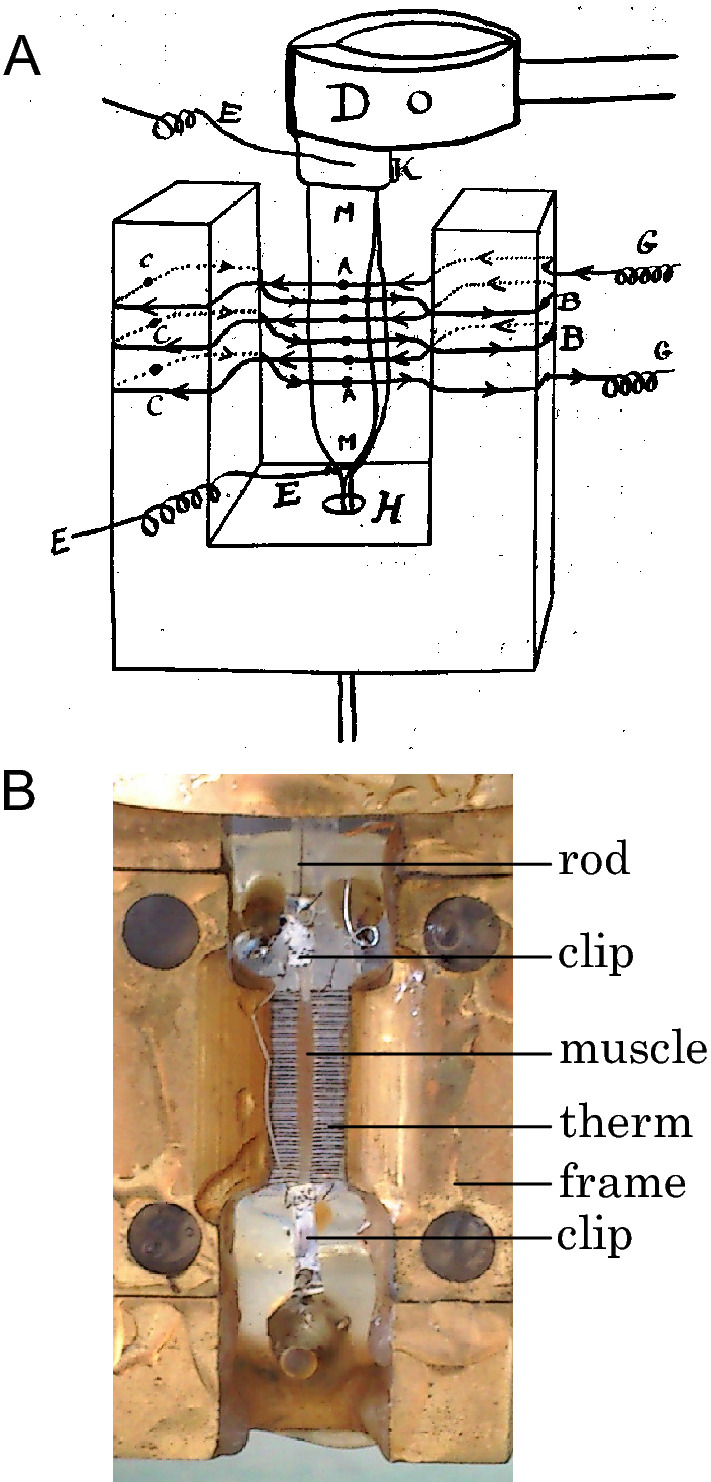
Thermopiles used to measure changes in muscle temperature. **A** Diagram of thermopile used by A. V. Hill (1913b). The thermopile lies between a pair of sartorius muscles (labelled “M”). The muscles cover the active thermocouples (**A**) and the reference thermocouples (**B** and **C**) are embedded in an ivoride frame. The thermocouples were made by soldering iron and constantan wires together. **B** A modern thermopile with vacuum-deposited antimony-bismuth thermocouples used for measuring heat production in bundles of fibres from mouse skeletal muscles (Barclay 2015b). The reference couples are embedded in a gold-coated brass frame. The muscle fibres are held in aluminium clips. The upper clip is attached by a rod to an ergometer

A.V. Hill, then at Cambridge University, entered the myothermic scene in 1910, using a thermopile-galvanometer system made by Blix (Hill 1910, 1911). In 1911, Hill visited Karl Bürker (Tübingen, Germany) to learn how to make thermopiles; Bürker was a creative designer and constructor of thermopiles, producing a range of different configurations in an effort record all the heat produced by a muscle (Bürker 1908; Hill 1913b). Some of his more conservative designs are recognisable as the predecessors of those used for the next 70 years (Fig. 2). During his visit, Hill also met the physicist Freidrich Paschen, who showed Hill how to make moving-coil galvanometers suitable for use with thermopiles (Hill 1959). Thereafter, starting with thermopiles based on Bürker’s, Hill commenced what was to be a 40 year program of improvements in every aspect of thermopile and galvanometer design. Hill applied a rigorous, quantitative approach to developing heat measuring equipment and worked on increasing sensitivity, reducing response time, improving calibration methods (required to convert muscle temperature changes to heat production) and minimising non-uniformities in muscle temperature.

Hill thought that the heat measurements made by Heidenhain and Fick were insufficiently reliable to provide evidence strong enough to reject the elastic theory. Hill carried out several studies that included examination of aspects of the elastic theory (Evans and Hill 1914; Hill 1913a; Hill and Hartree 1920). From his evidence, Hill agreed with Blix that isometric contractions produced more heat than those with shortening. However, these experiments were unconvincing, often involving manipulation of multiple, most likely interacting, variables and comparisons which were unlikely to distinguish between elastic or alternative mechanisms. In retrospect, Hill stated that a major impediment in these studies was that ideas of what was expected were unclear (Hill 1965b). In a positive step, Hill had settled on using the frog sartorius muscle because it had straight fibres and was thin. The straight fibres provide accurate measurements of muscle work output and the thinness provided rapid thermal equilibrium with the thermopile and reduced limitations to diffusive O_2_ supply throughout the muscle cross section (Hill 1928).

## Fenn’s test of the elastic model

In 1922 and 1923, Wallace O. Fenn, an American research fellow working in A. V. Hill’s laboratory in the UK, carried out an extensive series of experiments to test the idea that energy output as work and heat depended only on initial muscle length. Hill suggested these experiments to Fenn and provided the equipment (Fenn 1923). The Introduction to Fenn’s (1923) paper is evidence that Hill and Fenn had clarified what would be expected if the elastic theory was correct and Fenn (1923) summarised the view of adherents of the elastic theory thus,

*The amount of elastic potential energy which could be recovered as work … had no relation to the total energy liberated.*

**Fig. 3:**
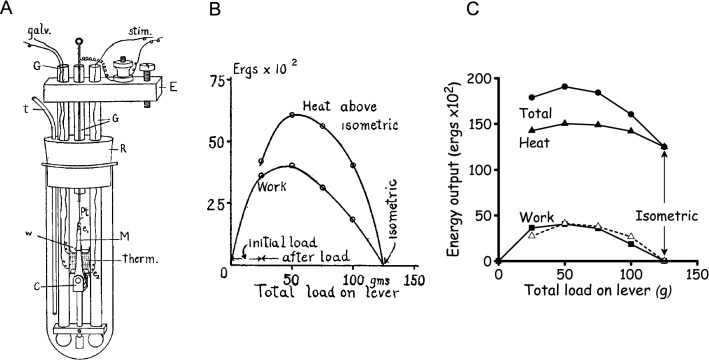
**A** Fenn’s chamber for measuring muscle heat production. The thermopile (labelled “Therm”) consisted of silver-constantan thermocouples made by electroplating (Wilson and Epps 1919). The active couples lay between two sartorius muscles (“M”) and the reference couples were in thermal contact with paraffin-filled glass tubes which also served as conduits for leads from the thermopile to the galvanometer. The thermopile and muscle were enclosed in a glass test tube with a rubber stopper (R); this was immersed an isothermal bath. There was no solution in the test tube when heat measurements were made to ensure that all the heat produced by the muscle went to the thermopile. From Fenn (1923), used with permission from John Wiley and Sons.** B**. Fenn’s illustration of the extra heat produced, above the isometric heat, when the muscle performed work. The muscle performed afterloaded contractions and the load on the lever is shown on the x-axis; light loads correspond to fast shortening and heavy loads to slow shortening. Energy output is in 10^2^ ergs (= 10 μJ). From Fenn (1923), used with permission from John Wiley and Sons.** C**. The data in B redrawn to correct the heat calibration error in Fenn’s original data and to illustrate the absolute heat (filled triangle) as well as the extra heat above isometric (triangle, dashed line) produced when doing work (filled square). The total energy output (filled circle) is the sum of the heat and work output. The notable feature is that isometric contraction produced less heat and less total energy than any contraction involving performance of work, a finding that was not consistent with the elastic theory

Fenn’s experiments were designed to determine the relationship, if one existed, between the total energy liberated, as work and heat, and the amount of work performed. This clarity of purpose was a major advance of Fenn’s work over the earlier studies. Furthermore, his experiments were more systematic than much of the earlier work. Fenn used Hill’s latest muscle heat measuring system (Hill and Hartree 1920) (Fig. 3A). The heat recordings were still quite slow, compared to the time course of muscle force production. For example, with frog muscle at close to 0 ºC, twitch contractions, which were commonly used at that time, were complete in ~ 0.5 s. In response to a heating pulse of similar duration, the galvanometer output took ~ 5 s to reach a peak. So, again, the records gave no information about the time course of heat production; instead, only the cumulative heat produced up to a time some seconds after a contraction was measured (Hill 1965b). Consequently, the differences in heat production between isometric and working contractions had to be determined by subtraction of two large and similar quantities. Many years later, it was also discovered that, despite Hill and Fenn’s best efforts, there was a substantial heat calibration error; the calculated heat values were ~ 1.5-times too large (Hill and Woledge 1962) but this does not affect the conclusions.

Fenn employed both a traditional lever system to perform afterloaded isotonic contractions and also an “inertia lever”, based on a device used by Fick (Hill 1920). With an isotonic lever, the muscle raised the load when its force generating capacity exceeded the load and then it lowered the load during relaxation. When the load was lowered an extra amount of heat, equivalent in magnitude to the work done, was produced. The total heat produced was then the sum of the heat generated by the muscle and the thermal equivalent of the work done. Hill, however, was not convinced that all the heat produced from lowering the load could be measured. The inertia lever allowed the muscle to relax against a very low load so that the heat output was measured directly rather than by subtraction from the total energy output as with the isotonic lever. Fenn’s textual clarity (see next paragraph) did not always extend to the tabulated data: confusingly, in his papers Fenn variously labels as “heat” that measured using the inertia lever and also that from afterloaded contractions which included the heat produced when the load was lowered (thus really total energy output, heat + work), making some of the tabulated data difficult to understand (Hill 1965b).

Fenn published his results in two papers (Fenn 1923; 1924), with the most important results in the first of these. The papers are notable not only for their scientific contribution but also for the clarity with which they are written, with clear expression of ideas underlying the experiments and lucid conclusions. It was also notable that Fenn used graphs to illustrate his results at a time when this was not common (tabulated data were more common). Fenn consistently observed that the total energy output was not independent of load; rather, more energy was liberated from muscles when they performed work than in an isometric contraction (Figs. 3B and C). Additionally, the extra energy was not just the work performed, additional heat was also produced. He found similar results for twitches and short tetani and using afterloaded isotonic contractions and the inertia lever. Fenn (1923) gave a concise conclusion.

*Whenever a muscle shortens upon stimulation and does work in lifting a weight, an extra amount of energy is mobilised which does not appear in an isometric contraction. Hence less energy is liberated in an isometric contraction than in any contraction in which the muscle is allowed to shorten.*

A striking aspect of the study from a modern perspective is that there was no form of statistical analysis available; the design of the experiments certainly lends them to multivariate analysis. In the second paper Fenn addressed the implications of his results for ideas about how muscle works, explicitly rejecting the elastic theory. Fenn stated that although a stimulated muscle might produce considerable tension, it may still possess little elastic energy, and thus the energy required to shorten and perform work had to be liberated as the shortening proceeds (Fenn 1924). Fenn gave full acknowledgement to the nineteenth century European researchers, particularly Fick, who had come to the same conclusion, albeit with less convincing experimental evidence.

## Consequences of Fenn’s paper

In 1923, Hill was awarded the Nobel Prize for the previous year and in his Nobel speech, given shortly before publication of Fenn’s 1923 paper, he briefly described Fenn’s results. He concluded,

*We are studying here the curious power which a muscle possesses of adapting its liberation of energy to the work it has to do. The subject is new and results are only now beginning to appear, but it is certain that we are dealing with a very fundamental property of the muscular machine.*

(Hill 1965a).

### Continued development of the elastic theory

Despite those sentiments, Hill and those working in his laboratory not only continued to use the elastic model as a framework for interpreting experiments but further developed it, incorporating a viscous element (Hill 1922). The new idea was that the solution within muscle cells had to be forced through some sort of structural network when the muscle shortened and that this provided a resistance to shortening, giving rise to a system with mechanical behaviour analogous to that of a damped spring. Overcoming the viscous resistance required some of the elastic potential energy which was converted into heat rather than work. The viscous-elastic model could account quantitatively for a range of phenomena (e.g. effects of shortening on force and work output and why measured efficiency was lower than that predicted from the supposed elastic potential energy) in both human (Hill 1922) and isolated frog muscles (Gasser and Hill 1924). Levin and Wyman (1927) further elaborated the model to account for their observations on “tension-length curves” (which would now be described as “work loops”) to consist of both a damped elasticity (i.e. the component affected by muscle viscosity) in series with an undamped elasticity. This model could account for the curved work-shortening velocity curves they measured with muscles from many different animals. These curves were actually the same as force–velocity curves and were the first of these to be measured.

**Fig. 4:**
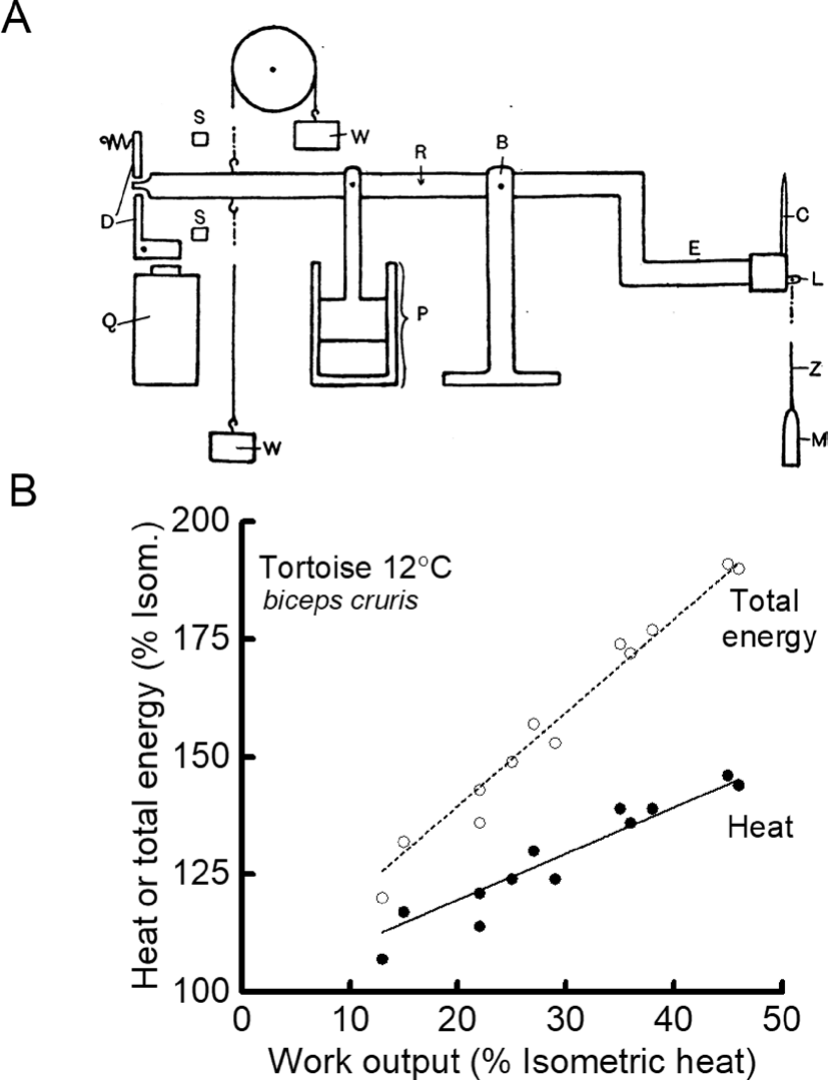
Wyman’s measurement of heat and work output. **A** Wyman (1926) developed a lever system that allowed precise control of the timing, amplitude and velocity of muscle length changes and as an output produced plots of tension as a function of length; the area enclosed by the curves was the work done. The muscle (M) was attached to a rotating beam (R) the velocity of which was controlled by weights (W) and a damping piston (P). From Wyman (1926), used with permission from John Wiley and Sons.** B**. Relationship between heat output and work done during shortening (solid symbols) and between total energy output (work + heat; open symbols) and work done for tortoise skeletal muscle. To account for difference in size amongst muscles, all energy quantities for each muscle were normalised by isometric heat output. Total energy output showed a linear dependence on work done; the original elastic theory predicted that the two variables would be independent. Wyman proposed that part of the elastic potential energy not used for work was reabsorbed during relaxation accounting for the relationships. Drawn from data in Table 2, Wyman (1926)

Experiments designed to repeat Fenn’s work were also carried out. Jeffries Wyman (1926), a doctoral student working in Hill’s lab and using a new inertia lever design (Fig. 4A), found heat production by tortoise muscle (used because its slow contraction and heat production kinetics might compensate for the slow response of the heat recording apparatus) was greater in a contraction with shortening than in an isometric contraction (Fig. 4B) but less in a contraction with stretching than one with shortening. These results were similar to those of Fenn (1924) and Fick (1881). Whereas those authors had concluded that the results indicated that physiological processes accounted for the heat and work output, Wyman produced what now appear rather contrived arguments—a variable fraction of potential elastic energy not used as work was reabsorbed during relaxation rather than appearing as heat—to support the idea that all the thermal changes could be explained by processes that were physical (i.e. dissipation of stored potential energy and effects of viscosity) rather than physiological. In 1928, Hill and Hartree repeated Fenn’s experiments on frog muscle and surprisingly found that whereas they could replicate Fenn’s results for tetanic contractions they could not replicate his observation of extra heat in twitches (Hartree and Hill 1928a).

Hill concluded that the only convincing way to address the question of whether additional energy was mobilised as shortening occurred was to improve the speed of the thermopile/galvanometer system so that the time course of heat production within a short tetanus (e.g. < 5 s) could be measured (Hill 1965b). This task was not trivial, ultimately requiring ways to make thermopiles with much finer thermocouple wires to reduce the contribution of the thermopile to overall heat capacity, without unduly increasing the electrical resistance (which would result in a smaller galvanometer deflection for a given amount of heat), and increasing the response speed of the galvanometers. As an interim measure, Hill’s long-term collaborator William Hartree developed a numerical analysis (which would now be called a deconvolution) to extract time course information from heat records made using inherently slow measuring apparatus (Hartree 1924). The extent to which time course information could be retrieved was limited but Hartree calculated that more heat was produced during shortening than at the same time in an isometric contraction but, conversely, less heat was produced during relaxation after shortening so that the overall heat production was the same in both cases (Hartree 1925). This puzzling result must have confirmed Hill’s idea that recordings of heat output with high temporal resolution were required to understand what performing work did to heat production. The numerical analysis, performed by hand, was taxing: Hill (1949b) recounted that Hartree estimated that he had written down between 10^7^ and 10^8^ figures when doing his muscle heat analyses!

In his 1965 book, Hill lists 10 papers published between 1925 and 1932 that had attempted to repeat or expand upon Fenn’s results but with little success. Hill summarised the post-Fenn attempts to confirm the 1923 results.

*In *Hill (1925)* “a possible mechanism of the Fenn effect” was discussed, but this lead nowhere. In 1925 Hartree attempted to analyse the course of the heat production during a contraction with shortening and work; but the results are doubtful because of the absence of a protecting region in the thermopile, and he did not realise how much work was done in an “isometric” contraction. In *Hartree and Hill (1928b)* the “Fenn effect” was clearly confirmed in tetanic contractions but not in twitches; the failure to do so was probably due to an error in the heat calibration. And so on.*

(Hill 1965b; p. 148).

Despite Hill’s rather pessimistic view (in retrospect, he felt that a great amount of work had been wasted due to his own inability to discard the elastic model), there was now a considerable body of information indicating that, even accepting the technical limitations, it was unlikely that the predictions of the elastic or viscous elastic model were correct. Furthermore, in successive heat studies from 1925 to 1928, Hill and his colleagues had made important developments of the heat measuring equipment and methods of analysis of heat records and these would eventually contribute to the system that was to settle the debate as to whether the performance of work was powered by energy from previously stored elastic energy or by energy from chemical reactions linked to the action of doing work.

## Fenn produces more evidence against elastic theories

In the decade after the Hartree and Hill (1928a) paper, Hill’s attention moved away from what he had termed “the Fenn effect” (Hill 1925). In 1935, Fenn returned to the discussion, publishing a provocative paper in which he produced an independent line of evidence to further support his contention that the elastic theories were untenable. Fenn and Marsh (1935) proposed an experimental test based on a viscous elastic model of muscle mechanical behaviour suggested by Levin and Wyman (1927). That model consisted of an undamped, non-contractile elasticity in series with a damped elasticity that represented the contractile element. Levin and Wyman stated that the damped component behaved in a viscous manner. Fenn and Marsh recognised that the properties of the damped or contractile element could be revealed by maintaining a constant force during shortening; in that case, the length of the undamped elastic component is constant and the shortening must reflect the properties of the contractile element. The viscous elastic model predicted that contractile element’s shortening velocity would decrease linearly with increasing load. Using afterloaded contractions to keep the force constant during shortening, Fenn and Marsh determined the force–velocity properties of cat and frog muscles. The force–velocity relationships were not linear but rather had the now familiar curved shape, indicating that muscles could not have a contractile element that behaved in a viscous-elastic fashion. In a superb and forthright summary, Fenn and Marsh (1935) wrote,

*It was shown by Fenn [1924] in Hill's laboratory that in order to shorten and do work a muscle must liberate an extra amount of energy more or less in proportion to the work done in shortening. This fact was held to cast doubt upon the theory that a muscle is simply an elastic body. In spite of this objection subsequent authors have continued to explain phenomena observed in shortening muscles on the basis of simple mechanical models. The present experiments show again that these simple models are inadequate for the purpose, and it seems most likely that they are inadequate for the same reason that muscle is not an elastic body.*

## A. V. Hill’s ultimate response: empirical confirmation of Fenn’s ideas

**Fig. 5:**
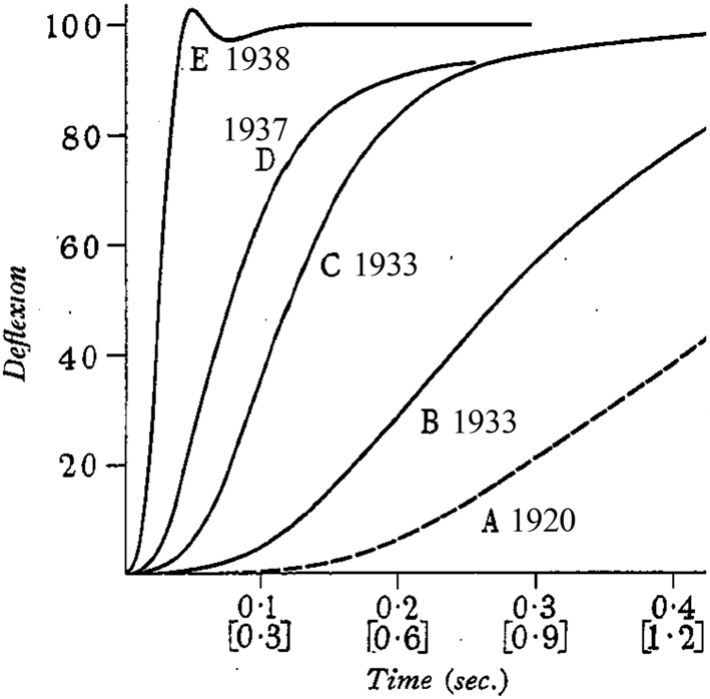
Improvements in the response time of myothermic apparatus, 1920–1938. The traces show the response of the thermopile-galvanometer systems to a step increase in temperature of the active thermocouples. The year that each system was developed is shown. The lower x-scale applies to the 1920 system and the upper x-scale to the other years. In particular note the improvement in response achieved between 1937, when the preliminary report of Hill’s new experiments appeared, and 1938 when the full paper was published. From Hill (1939), used with permission from IOP Publishing

In 1937, Hill published a methods paper that marked the culmination of 25 years of development of myothermic apparatus that started with his first Bürker-derived system (Hill 1913b). Hill (1937) described a new fast-responding, low heat capacity thermopile linked to a fast but sensitive galvanometer. With this system, the heat recording step response was complete in ~ 200 ms, a 25-fold increase in speed since Fenn’s studies (line “D”, Fig. 5). The thermopile was equipped with a “protecting region”, an extra section of thermopile under the end of the muscle at which the lever was attached. This was not part of the temperature recording circuit but served to ensure that heat loss from the muscle into the thermopile was the same along the part of the muscle over the recording region at the start of the contraction and along the length of muscle that moved onto the thermopile during shortening. This simple measure solved the long-standing problem that increases in heat during shortening may have been due to warmer sections of muscle moving onto the thermopile (Hill 1913b). Hill had also further developed the deconvolution analysis to correct for both lag in heat records due to the time taken for heat to move from the muscle to the thermocouples and for heat lost from the muscle through the thermocouple wires. Finally, a much-improved heat calibration method had been developed.

In a report to The Physiological Society, UK, in May 1938, Hill (1938a) gave a preliminary description of experiments, performed using the new apparatus, that unequivocally confirmed Fenn’s conclusions, unveiled Hill’s mathematical description of the force–velocity relation and illustrated that muscle might usefully be considered as consisting of a contractile element, with the observed force–velocity properties, connected in series with an elastic element. This work was published in full in October in a paper entitled *The heat of shortening and the dynamic constants of muscle* (Hill 1938b). Hill also presented the results to the Physical Society in November and he recommended (Hill 1965b) the published version of that presentation (Hill 1939) as “more readable than the original paper”. An example of Hill’s continual pursuit of better methods was that between the work described in May and the full paper in October, an even faster galvanometer system had been developed, giving the system a step response time of just 50 ms (Fig. 5) (for a description, see Hill 1939).

**Fig. 6:**
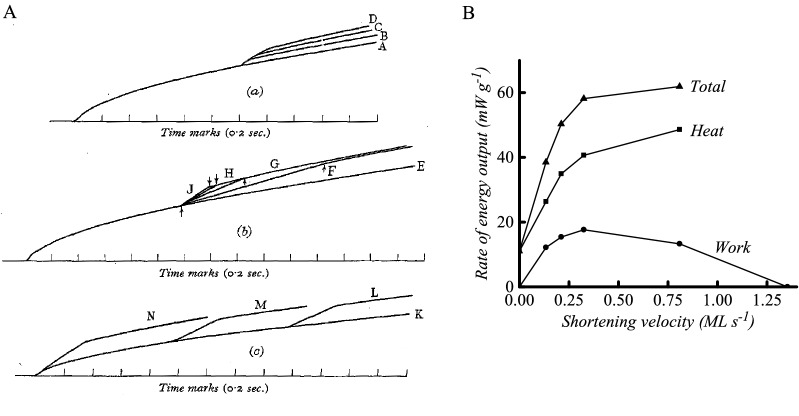
Summary of Hill’s (1938a, b) findings. **A** Records of time course of heat production. Records labelled A, E and K are from isometric contractions; the other records are from contractions with work performed during a period of isotonic shortening. The onset of shortening is indicated by the upwards deviation in the heat record; that is, rate of heat output increased when shortening started. Records in (a), shortening different distances at a constant load; (b), shortening constant distance under different loads; (c), shortening constant distance with same load, starting at different times. The amount of heat produced was (a) proportional to the distance shortened, (b) independent of load and (c) independent of time of start of shortening. Records from frog sartorius muscle, at 0 ºC. Figure reproduced from Hill (1939), used with permission from IOP Publishing. **B** Relationship between rate of energy output and velocity of shortening. Symbols, experimental points; created using data from Hill (1938b) (Hill’s Fig. 9, line “F” and information in figure legend)

With this apparatus, Hill concentrated on recording muscle heat production in tetanic contractions in which the muscles were initially held isometric and then at a specified time were allowed to shorten a set distance against a constant load. In planning the experiments, Hill adopted the constant force protocol described by Fenn and Marsh (1935) so that the observed force–velocity properties could be attributed solely to the contractile element. The results of the experiments were clear: the rate of heat production increased abruptly above the isometric rate when shortening started and decreased when shortening ended (Fig. 6A). The amount of heat produced was largely determined by how much the muscle shortened and was not greatly affected by the load nor by the time within the contraction that shortening started (Fig. 6A). In Fig. 6B a summary is shown of the dependence of rate of energy output on the velocity of shortening for one muscle. Work output is 0 in an isometric contraction and when shortening at maximal velocity (*V*_max_) and rises to a maximum at about 25% *V*_max_. The rate of heat output increased with shortening velocity, as did the rate of energy output, as both heat and work. This was a convincing demonstration that extra energy was mobilised as the muscle shortened and performed work and that the extent of the mobilisation depended primarily on how much shortening occurred. Although this conclusion was the same as Fenn’s, the heat records with the greatly improved time resolution provided unequivocal evidence. And thus, after almost 100 years, the elastic theories were finally extinguished.

Much of Hill’s discussion about the energy output during shortening focussed on what he called “shortening heat”, which was the heat in excess of the underlying isometric heat. This idea was problematic, with considerable discussion over what constituted the appropriate baseline (Hill 1964; Mommaerts 1970; Rall 1982). Hill (1939) did suggest that it might be more accurate to think of the increased rate of heat output as due to a change in the rate of all the heat producing processes involved in generating force rather than just the addition of an extra component, a view that is closer to our current understanding than the shortening heat concept. However, the scope for mechanistic thinking in 1938 was limited, given that the scheme of chemical reactions occurring in muscle was not known, the distinction between the events of activation and those of force generation had not been elucidated and the uncovering of the structural basis of force generation was still 20 years in the future.

The 1938 paper wasn’t Hill’s last word on the topic. After time spent on war-related activities (for descriptions, see Barclay and Curtin 2022; Katz 1978; Rall 2017), Hill returned to science in the late 1940s. Following further developments of his heat-measuring apparatus (Hill 1949a), Hill showed that more energy, as both heat and work, was produced in twitches with shortening (Hill 1949b, 1953), correcting an earlier anomalous result (Hartree and Hill 1928a). Further, when Hill and others repeated his 1938 experiments with improved apparatus, the relationship between shortening heat and distance shortened was not as simple as proposed in 1938 but instead showed a degree of load-dependence (Hill 1964). This result does not diminish the idea that performance of work and production of heat are related but did make the relationship between the mechanical performance and energy output less straightforward than suggested in 1938.

## Interpreting Fenn and Hill’s experiments

There was limited scope for insights into the basis of the increase in heat output that accompanied the transition from isometric contraction to work generation, particularly in 1938 but also in 1964 when Hill completed his revisionary experiments on heat output from muscles performing work. However, in the 20 years after the 1964 paper, several key aspects of muscle energetics were elucidated. These are briefly summarised in the following paragraphs; Rall (2018) gives a comprehensive account of these and many other aspects of muscle physiology.

### Sequence of chemical reactions underlying contraction

Although it had been recognised since at least the 1840s that chemical reactions must ultimately account for muscle heat production, knowledge of the nature of the reactions that occurred as a muscle contracted remained fragmentary prior to the 1960s. For 30 years after the discovery of ATP in muscle (Fiske and Subbarow 1929; Lohmann 1929), there was only indirect evidence that it played a primary role in contraction. The only consistent chemical change seen to occur in muscle within the time course of a short contraction was a decrease in PCr concentration (for reviews, see Mommaerts 1969; Needham 1950). In 1962, Davies and colleagues provided direct evidence that ATP splitting was the primary energy-providing reaction in muscle (Cain and Davies 1962; Cain et al. 1962). They first showed that under normal circumstances in frog muscle, PCr was broken down in proportion to the amount of work a muscle performed. They then inhibited the creatine kinase (CK) reaction (Reaction 2, Table 2) using dinitrofluorobenzene (DNFB), preventing phosphorylation of ADP by PCr; in response to stimulation, the muscles did contract but there was no decrease in PCr concentration. The concentration of Pi increased, as in untreated muscle, but now ATP concentration decreased and the concentrations of ADP and AMP increased (Reactions 1 and 4, Table 2). This established the currently accepted reaction scheme: ATP splitting provides the energy for contraction and ADP formed in that reaction is rapidly rephosphorylated at the expense of PCr in the cytoplasm (i.e. the CK reaction; Reaction 2, Table 2). PCr is subsequently regenerated using ATP in a reversal of the CK reaction (Reaction 6), that occurs in the mitochondrial intermembrane space proximal to the adenine nucleotide translocase and is catalysed by a different version of CK, using ATP produced by oxidative or anaerobic processes (Reactions 7 and 8, Table 2).

**Table 2:** Energetically important reactions that can occur during a brief contraction

1 2	ATP hydrolysis CK reaction	${\text{ATP}} \to {\text{ADP}} + {\text{Pi}}$ ${\text{PCr}} + {\text{ADP}} \to {\text{ATP}} + {\text{Cr}}$	AM-ATPase & ion pumping ATPases Fast ATP regeneration
3	Net initial reaction	${\text{PCr}} \to {\text{Cr}} + {\text{Pi}}$	Usual observed chemical change
4 5	Myokinase reaction	$2{\text{ADP}} \to {\text{ATP}} + {\text{AMP}}$ ${\text{AMP}} \to {\text{IMP}} + {\text{NH}}_{{\text{3}}}$	In vivo, typically occur only to small extent; more prominent in isolated muscle with CK inhibition or PCr depletion
6	CK reaction, reversed	${\text{Cr}} + {\text{ATP}} \to {\text{PCr}} + {\text{ADP}}$	Regeneration of PCr using ATP
7 8	Anaerobic glycolysis Ox. phosphorylation	${\text{Substrate}} + {\text{ADP}} \to {\text{ATP}} + {\text{La}}$ ${\text{Substrate}} + {\text{ADP}} + {\text{O}}_{{\text{2}}} \to {\text{ATP}} + {\text{CO}}_{{\text{2}}} + {\text{H}}_{{\text{2}}} {\text{O}}$	Substrate-level ATP synthesis O_2_-coupled ATP synthesis
9	Overall net reaction	${\text{Substrate}} + {\text{O}}_{{\text{2}}} \to {\text{CO}}_{{\text{2}}} + {\text{H}}_{{\text{2}}} {\text{O}}$	

*AM-ATPase* actomyosin ATPase; *CK* creatine kinase; *La* lactic acid; *Ox.* oxidative

With the demise of the elastic theory, it was clear that the source of the heat and work output from contracting muscles was the chemical reactions that occur as contraction progresses but the question remained as to which reactions were represented in the heat output. The “energy balance” experiments of the 1970s and 1980s examined the quantitative relationship between heat produced and the extent of the underlying biochemical reactions (for reviews, see Barclay 2015a; Barclay and Loiselle 2021; Homsher 1987; Woledge et al. 1985). The principle of the investigations was to compare the amount of heat actually produced to that expected based on the measured extent of all the reactions that have occurred. If the measured and predicted heats align, then it can be concluded all the relevant reactions are known. The information from those experiments provided much of our current knowledge of biochemistry in living muscle as depicted in Table 2.

Of relevance to understanding Hill’s 1938a, b heat records, the energy balance experiments showed that most of the heat produced during a short isometric contraction was due to the net breakdown of PCr (Reaction 3, Table 2) (Curtin and Woledge 1979; Homsher et al. 1979). At the start of a contraction there is a transient production of heat produced by Ca^2+^ binding to troponin C and, in some muscles (e.g. muscles of amphibians, fish and fast-twitch muscles of, at least small, mammals), parvalbumin in the cytoplasm. Once this is complete, the time course of heat production reflects the time course of PCr breakdown and, therefore, of ATP turnover (Curtin and Woledge 1979; Homsher et al. 1979). This is important because Hill in his 1938 and 1964 studies used a protocol in which shortening typically started only after several seconds of isometric contraction (Fig. 6). Therefore, it is reasonable to assume that Hill’s measurements of energy output during the transition from isometric to shortening contraction accurately reflected changes in the rate of ATP hydrolysis. It should be noted that an advantage of using low temperatures, as in the frog muscle studies, was that the oxidative reactions that regenerate PCr occurred so slowly, requiring 30 min to be complete, that heat from those reactions did not contribute to the heat produced during a contraction lasting only a few seconds.

### Activation heat

The discovery of the role of Ca^2+^ in the activation of muscle (for a review, see Ebashi and Endo 1968) suggested that removal of Ca^2+^ from the fibre by the sarcoplasmic reticulum’s Ca^2+^ pump contributes to energy turnover and thus heat production. The sliding filament theory (Huxley and Hanson 1954) provided a basis for partitioning heat production into force-dependent and force-independent (or activation) components. Comparison of the heat produced during an isometric contraction at full overlap between the thick and thin filaments with that when muscle length had been increased sufficiently to eliminate filament overlap revealed that force-independent, or activation, processes account for ~ 30% of the heat produced during an isometric tetanus (Homsher et al. 1972; Smith 1972). This component is mainly due to ATP splitting by the Ca^2+^ pump, with a small, but as yet poorly quantified, part likely to be due to the sarcolemmal Na^+^–K^+^ ATPase (Barclay et al. 2007). Heat from the Ca^2+^ binding reactions mentioned above may also contribute to activation heat, depending on the measurement protocol used.

The relevance of the partitioning of heat output between force-dependent and activation components for understanding Hill’s 1938a, b heat measurements is that the change in rate of heat output that occurred when shortening started and stopped must predominantly be due to changes in the force-generating processes rather than the activation processes.

### Cross-bridge theory of force generation

After the demise of the viscous-elastic models due to Hill’s 1938a, b papers, there was no convincing alternative model of how the contractile element generated force and work until 1957 when A. F. Huxley proposed a theory of force generation based on cyclic attachment of myosin cross-bridges to discrete binding sites on an adjacent actin filament. The cyclic nature of the process, first suggested by Needham (1950), is important to understanding the Fenn/Hill results in that cross-bridges undergo their attachment cycles, with ATP splitting in each cycle, even during isometric contraction. When the filaments slide past one another as muscles shorten during the performance of work, the cycling continues, albeit at an increased rate driven by the relative movement of the filaments (Huxley 1957). Therefore, in both isometric and working contractions, the force-dependent energy output (as heat and work) arises from the same process, the cycling of cross-bridges. In this scheme, there is no mechanistic justification to separate heat output into that occurring during isometric contraction and a distinct component occurring during shortening.

With the foundations of a defined reaction scheme and its link to heat output, the idea of activation heat and a cross-bridge-based contraction scheme, more recent experiments that cement the link between performance of work and energy turnover can be described.

## Biochemical equivalent of Hill’s 1938 experiments

If it is assumed that Hill’s records of the time course of heat and work output provided an accurate reflection of ATP turnover, then an obvious question is does the change in rate of energy output as described by Hill (1938b) have a biochemical equivalent? This was first addressed in several of the classical energy balance experiments. However, the time resolution possible with the methods of those experiments was limited and produced comparisons similar to those in Fenn’s studies. That is, it was found that muscles used more PCr when performing work than during an equivalent period of isometric contraction (Curtin et al. 1974; Homsher et al. 1984; Kushmerick and Davies 1969) but it was not possible directly compare the time courses of the energy output and chemical changes. Eventually, a different approach was used to record the rate of ATP breakdown with temporal resolution comparable to Hill’s 1938a, b recordings of heat and work output.

Permeabilised muscle fibres are an attractive preparation for probing fundamental aspects of muscle activation and contraction because the permeabilised sarcolemma allows the experimenter to control the composition of the solution surrounding the myofibrils. Mike Ferenczi and his colleagues were pioneers in using permeabilised fibres to study the fast ATP splitting events at the start of contraction, principally as a way to examine details of the cross-bridge cycle (Ferenczi 1986; Ferenczi et al. 1984). Central to this work was the ability to activate fibres rapidly using caged ATP, a non-metabolically active, photolabile compound from which ATP can be released by exposure to a brief light pulse. Fibres are first equilibrated in a solution containing caged ATP and sufficient Ca^2+^ to fully activate the fibre. Under those conditions, cross-bridges bind to actin but, in the absence of free ATP, cannot detach and cycle. When the ATP is released, cross-bridge cycling commences and the fibre contracts. Ferenczi et al. (1995) then incorporated into the fibre solution a fluorescent probe for inorganic phosphate (Pi) (Brune et al. 1994), one of the products of ATP hydrolysis (Reaction 1, Table 2). This provided a means to follow the time course of ATP splitting (He et al. 1997). The rapid activation was important so that force developed rapidly enough that the isometric and shortening phases could be completed before the Pi probe became saturated with Pi. An important difference between this preparation and the intact muscles used by Hill is that in the permeabilised fibres the only process consuming ATP was cross-bridge cycling; that is, there was no equivalent of the activation heat in the permeabilised fibres (He et al. 1997).

**Fig. 7:**
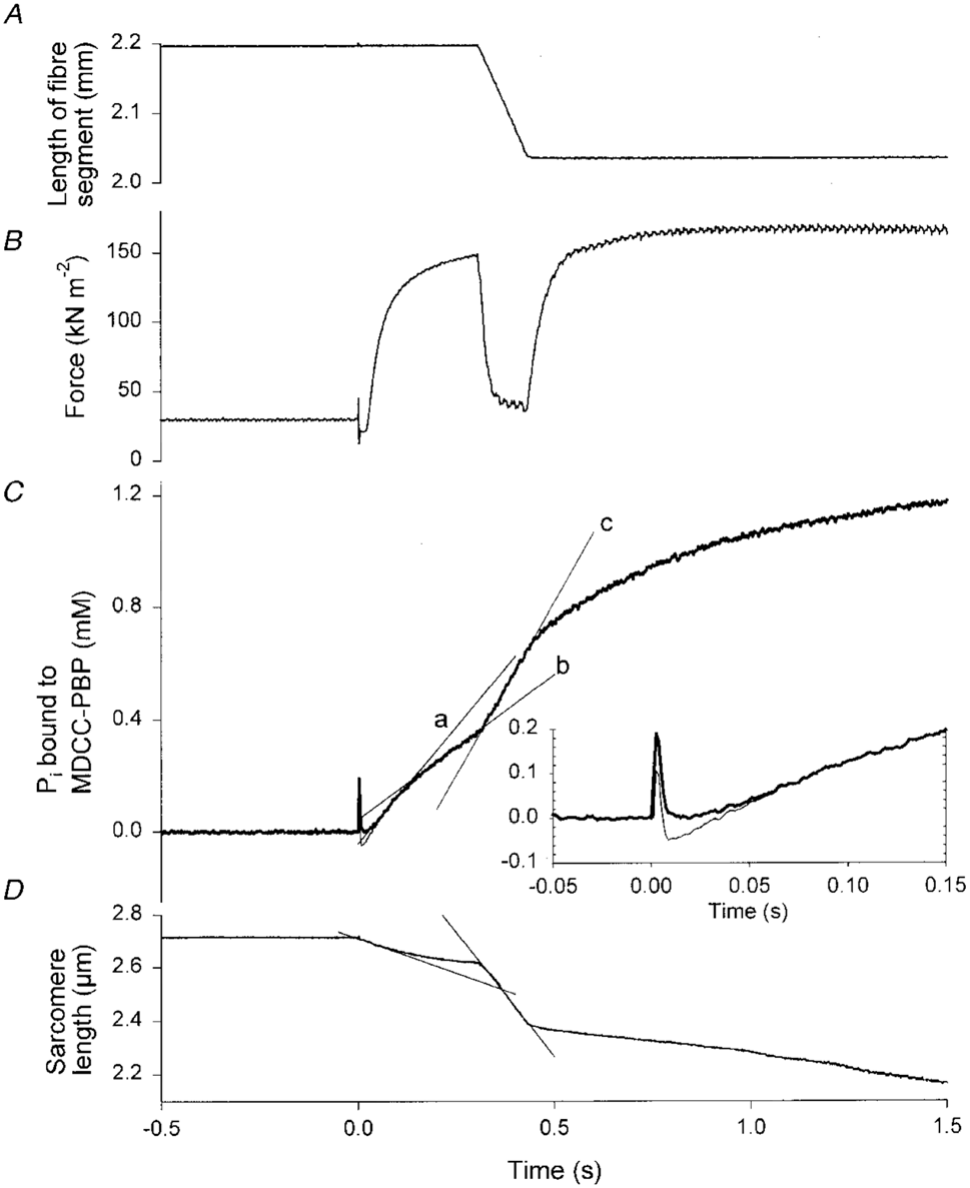
Rabbit psoas fibre at 12 ºC, activated by photolysis of caged ATP initiated by flash at time 0. After 0.3 s isometric contraction, muscle shortened at velocity of ~ 50% of the maximum shortening velocity, which produced a force of 30% of the isometric force (**B**). When shortening started, there was an immediate increase in the rate of Pi production (**C**), indicating more rapid ATP splitting. The rate stayed high throughout the time the fibre was shortening and then decreased once shortening stopped and isometric contraction resumed. From He et al. (1999) used with permission from John Wiley and Sons

He et al. (1999) measured the time course of Pi formation during a contraction protocol similar to that used by Hill (1938b); that is, an initial isometric phase followed by a period of shortening. In Fig. 7C, a record is shown of the time course of ATP splitting, as inferred from Pi release, in a rabbit psoas fibre following activation by release of ATP (He et al. 1999). For the first 0.3 s of contraction, the fibre was held isometric (Fig. 7A), although sarcomere shortening did occur (Fig. 7D). During this phase, there was a transient high rate of ATP splitting (indicated by slope of line “a”, Fig. 7C) which decreased to a steady rate (slope of line “b”). When the fibre started shortening, the rate of ATP splitting increased (slope of line “c”, Fig. 7C) and remained high while shortening continued. When the shortening stopped, the rate of ATP splitting decreased back to a similar rate to the pre-shortening isometric rate.

The time course of Pi formation is reminiscent of Hill’s heat records and it is reasonable to take this as confirmation that the change in rate of heat output at the onset of shortening in Hill’s experiments was due to an increase in the rate of ATP splitting by cross-bridges. The Ferenczi group’s experiments provide direct confirmation that a shortening muscle mobilises energy, from ATP splitting, as and when it is required to perform work, as proposed so clearly, but with limited evidence, by Fenn 76 years earlier.

## Is the effect of performing work on ATP turnover a general property of skeletal muscle?

**Fig. 8:**
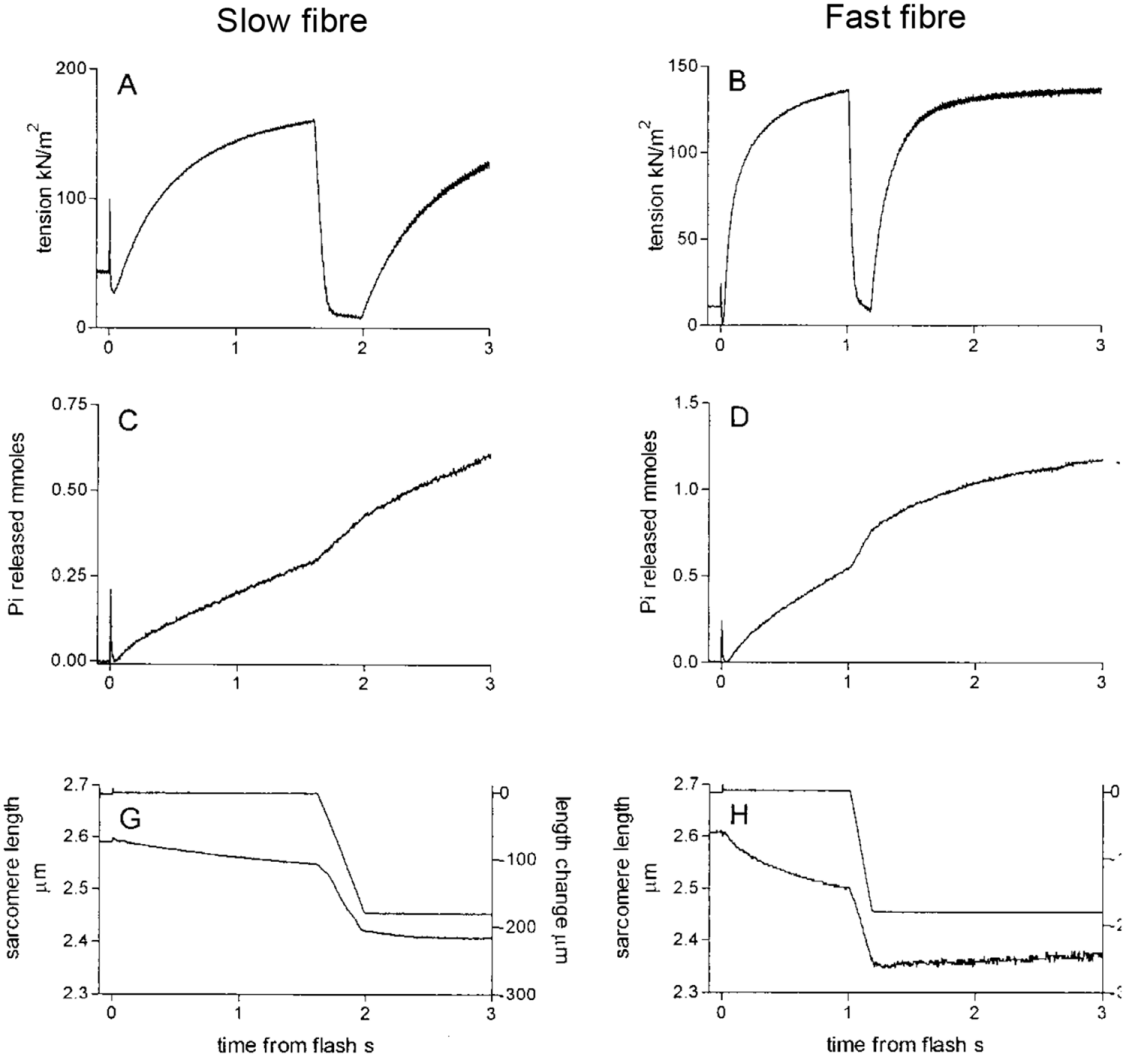
ATP splitting in human muscle fibres. Records showing time courses for fast and slow fibres of force output (panels **A** and **B**), Pi release (**C** and **D**) and sarcomere length (**G** and **H**). The higher rate of ATP splitting when shortening than isometric is apparent from the Pi release records for both slow fibres (**C**) and fast fibres (**D**). Reproduced from He et al. (2000) with permission from Elsevier

Data from frog sartorius (Hill 1938b) and rabbit psoas (He et al. 1999) already described in this article are consistent with the idea that ATP turnover alters dynamically depending on the mechanical loading encountered during a contraction. But is this a characteristic of all skeletal muscles? The abrupt increase in rate of heat production when shortening starts has been shown to occur in muscles from tortoise (Woledge 1968), fish (Curtin and Woledge 1991) and mice (Barclay et al. 1993, 2010b). Increased rate of Pi release has been observed not only for rabbit muscle but also for fast and slow fibres from human muscle (He et al. 2000). The human fibre study is important because all prior investigations had used muscles or fibres from small animals. However, the human muscle fibres exhibited faster ATP turnover during shortening than during isometric contraction, just as seen for muscles from other species (Fig. 8). In several experiments, the effect of shortening on rate of ATP splitting in permeabilised fibres was measured using an assay in which ATP hydrolysis is linked to the oxidation of NADH, a reaction which can be followed spectrophotometrically (Glyn and Sleep 1985). The temporal resolution of the NADH-based assay is usually not as good as that of the Pi probe (with the exception of Sun et al. 2001) but the overall effect of shortening on ATP use can be calculated. Using that approach, faster ATP hydrolysis during shortening has been demonstrated in rabbit psoas fibres (Potma and Stienen 1996; Sun et al. 2001) and in different fibre types of rat muscle (Reggiani et al. 1997).

Overall, it does appear that dynamic mobilisation of energy as it is required is a fundamental characteristic of skeletal muscle. It is worth recognising some differences between permeabilised preparations and intact muscle fibres. Permeabilised fibres are provided with simplified energy supply systems and the metabolic factors that limit ATPase activity in permeabilised fibres may differ from those in intact muscle (West et al. 2004). For example, although the Pi probe provides a direct view of ATP splitting, it also prevents the accumulation of Pi (He et al. 1999). In intact muscle, Pi accumulation slows the rate of ATP splitting (West et al. 2004). Conversely, although heat measurements are non-specific (i.e. heat may arise from multiple sources), when used with the protocol guidelines already mentioned, they provide a good index of ATP turnover subject to normal physiological constraints.

An indirect confirmation of the validity of the dynamic energy mobilisation concept is provided by biomechanical modelling studies of the movement of human limbs. In most multi-muscle models of limb movement, each muscle’s behaviour is modelled using an extended version of Hill’s two-component muscle model. That is, the contractile element is characterised by a force–velocity relationship which allows the muscle’s force output at any moment to be determined from the velocity at which the muscle is shortening or lengthening. Account is taken of the time course of activation, prior activity, etc (Winters 1990). Some of these models incorporate muscle energetics. In that case, the contractile element is also supplied with a velocity-dependent heat output (like that shown for frog muscle by the total energy output line in Fig. 6B). Like the force–velocity relation, this is the steady-state relationship determined in the same way as described by Hill (1938b) and scaled appropriately to human muscle (Tsianos et al. 2012; Umberger et al. 2003). The energetics predicted by such models align well with measured human energy output (Tsianos and MacFadden 2016; Tsianos et al. 2012; Umberger et al. 2003). It is particularly notable that even when using steady-state energetic profiles, these models can successfully predict energy use in rapid movements (Umberger et al. 2003). This emphasises that the energetic response of muscles to activity is rapid and dynamic. The success of such models indicates that the basic energetic properties obtained using isolated muscle preparations are applicable to complex real-world muscular activity.

## Cross-bridge basis of increased rates of energy output and ATP splitting

**Fig. 9:**
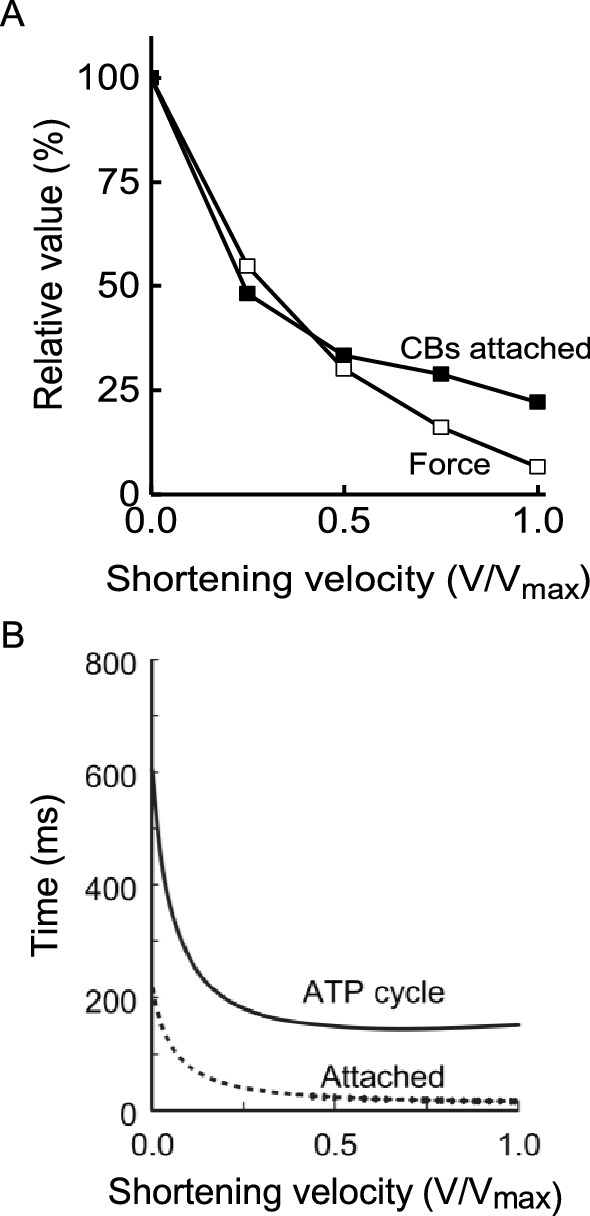
Cross-bridge properties during shortening.** A**. Variation with shortening velocity of force output (empty square) and number of attached cross-bridges (filled square). For shortening velocities < 0.5v_max_, the decline in force with velocity is all due to fewer cross-bridges being attached simultaneously. At higher velocities, the velocity-dependence of force reflects both fewer attached cross-bridges and lower average force per attached cross-bridge. **B** Variation with shortening velocity in duration of cross-bridge ATP splitting cycle and duration of attachment. Calculated from data for frog muscle contracting at 0 ºC. As velocity increases, the duration of the complete cross-bridge cycle decreases. The duration of the attached phase of the cycle decreases to an even greater extent than the complete cycle. From Barclay et al. (2010a), used with permission from Elsevier

Measurements of the rate of cross-bridge ATP splitting, determined from either Pi probe data or from energy output, can be used to gain insight to the changes in cross-bridge behaviour that produce the shortening velocity dependence of rate of ATP turnover. In a contracting muscle, cross-bridges go through a mechanical cycle involving attaching to a binding site on actin, generating force that promotes filament sliding and detaching. The mechanical cycle is linked to a biochemical cycle with, in its simplest form, a cross-bridge binding ATP prior to attachment and ATP hydrolysis and sequential release of the products Pi and ADP during the attached phase. During isometric contraction of frog muscle, between 30 and 40% of all cross-bridges are attached simultaneously (for explanations of how this can be estimated, see Barclay et al. 2010a; Linari et al. 2007; Offer and Ranatunga 2010). Cross-bridges that are not attached are presumably in the detached part of their cycle, repriming before attachment is again possible. When a fibre is actively shortening, fewer cross-bridges are attached than in an isometric contraction (Fig. 9A) (Barclay et al. 2010a; Caremani et al. 2015; Ford et al. 1985; He et al. 1999). This indicates that during shortening the repriming part of the cycle occupies a larger fraction of the full cycle than in an isometric contraction (Barclay et al. 2010a). However, the increased rate of ATP splitting indicates that the time to complete a full cycle has also decreased (Fig. 9B). Therefore, during shortening cross-bridges cycle faster and spend less of their cycle attached and the overall rate of ATP splitting (the product of cross-bridge cycling rate and cross-bridge concentration) is greater than during isometric contraction. The mechanism by which shortening influences the duration of cross-bridge attachment must involve rate constants for transitions between attached states that are sensitive to the mechanical strain experienced by the cross-bridge. Elucidation of the exact mechanism remains an active area of research (Caremani et al. 2015; Homsher et al. 1997; Linari et al. 2015).

## Conclusion

Fenn’s 1923 and 1924 papers are now viewed as a landmark in the development of ideas about muscle energetics. However, it would be fair to say that his work was not decisive; the limitations of the experimental methods made the results a less than reliable basis for the final interpretation. Fenn’s clear expression of his conclusions probably transcended the results of his experiments. But he found independent support for his ideas from his force–velocity experiments in 1935 and, perhaps most importantly, his work stimulated further research. Although Hill’s prolonged adherence to the elastic theory was a major element in the delay in accepting Fenn’s ideas, he deserves credit for recognising what was required to settle the matter and persisting for 15 years with technical developments to the point where he could show that his earlier ideas were wrong and that Fenn was right.

**Fig. 10:**
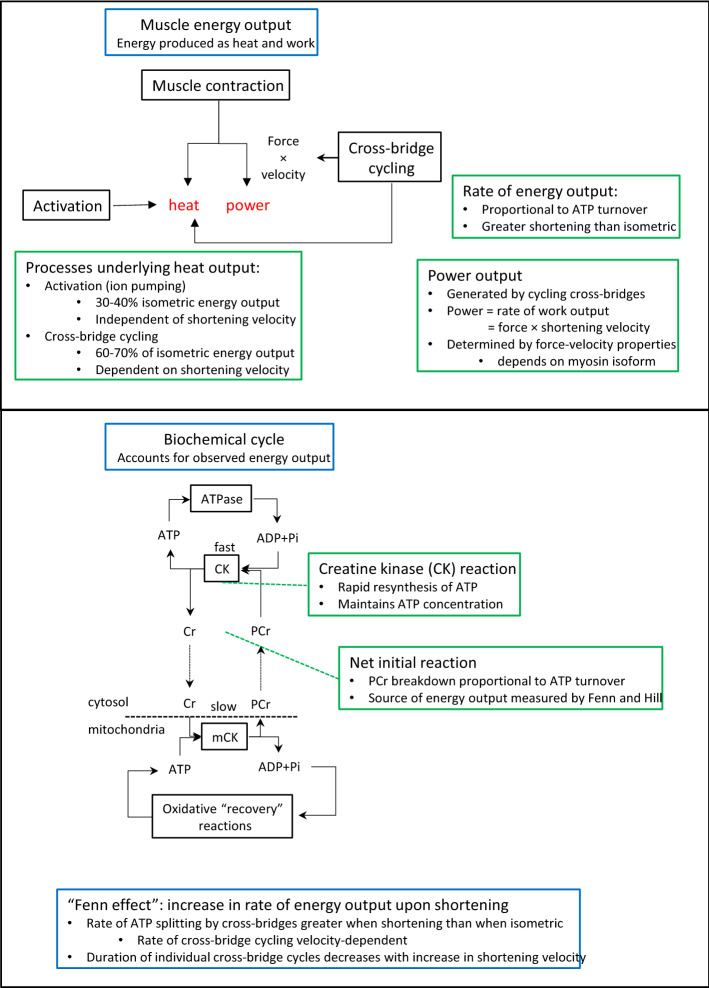
Summary of the key concepts of skeletal muscle energetics that underlie the difference in rate of energy output during isometric and shortening contractions. The upper panel depicts muscle energy output, as heat and mechanical power. Cross-bridge cycling accounts for the mechanical output (force production and power generation) and also part of the heat production, including the shortening velocity-dependent component of the heat production. Activation processes (mainly Ca^2+^ pumping) also contribute to the heat output. The lower panel illustrates the biochemical underpinnings of the energy output. The energy obtained from ATP hydrolysis is the ultimate source of energy for ion pumping and cross-bridge cycling. ADP formed in that reaction is rapidly rephosphorylated at the expense of PCr. This keeps the concentration of ATP constant so there is no *net* energy output due to ATP hydrolysis. In the presence of O_2_, PCr is subsequently regenerated by oxidative processes. In the experiments performed by Fenn and Hill the low temperature used (0ºC) made oxidative processes so slow that there was no PCr regeneration within the time course of their measurements. Hence, the energy produced by the muscles, as heat and mechanical work or power, can be attributed to the net breakdown of PCr. The effect Fenn observed of greater heat output and work output when shortening than when isometric is consistent with more rapid cross-bridge cycling, and hence shorter duration cross-bridge cycles, in contractions involving shortening than in isometric contractions

To understand Fenn’s results, and those of Hill, required the surge in knowledge that occurred after Hill’s final work on the matter in 1964. That era provided knowledge of how force and work was generated in muscle and how muscle biochemistry was related to both heat measurements and the force-generating cross-bridge cycle. That knowledge provided a basis for understanding the “Fenn effect” as a reflection of accelerated ATP splitting by cross-bridges when filament sliding commenced, promoted by strain-dependent rate constants for steps within the attached phase of the cross-bridge cycle (Fig. 10). Assembling that mechanism can be considered as the final stamp of approval for Fenn’s ideas, a process 76 years in the making. Fenn’s bold pronouncement of the implications of his work for understanding muscle was ultimately proved right and marked out a key concept in muscle energetics:

*The rate at which energy is liberated from ATP hydrolysis by cross-bridges during the contraction is modulated by the loads encountered during the contraction.*

## References

[CR1] Barclay CJ (2015). Energetics of contraction. Compr Physiol.

[CR2] Barclay CJ (2015). A mathematical model of heat flow in a thermopile for measuring muscle heat production: implications for design and signal analysis. Physiol Meas.

[CR3] Barclay CJ, Curtin NA (2022). The legacy of A. V. Hill’s Nobel prize winning work on muscle energetics. J Physiol.

[CR4] Barclay CJ, Loiselle DS (2021). Historical perspective: heat production and chemical change in muscle, Roger C. Woledge Progress Biophys Mol Biol.

[CR5] Barclay CJ, Constable JK, Gibbs CL (1993). Energetics of fast- and slow-twitch muscles of the mouse. J Physiol.

[CR6] Barclay CJ, Woledge RC, Curtin NA (2007). Energy turnover for Ca^2+^ cycling in skeletal muscle. J Muscle Res Cell Motility.

[CR7] Barclay CJ, Woledge RC, Curtin NA (2010). Inferring crossbridge properties from skeletal muscle energetics. Prog Biophys Mol Biol.

[CR8] Barclay CJ, Woledge RC, Curtin NA (2010). Is the efficiency of mammalian (mouse) skeletal muscle temperature dependent?. J Physiol.

[CR9] Becquerel A, Breschet G (1835). Sur la chaleur animale. Ann Sci Nat.

[CR10] Blix M (1902). Studien über Muskelwärme. Skand Arch Für Physiol.

[CR11] Brooks GA (2012). Bioenergetics of exercising humans. Compr Physiol.

[CR12] Brune M, Hunter JL, Corrie JE, Webb MR (1994). Direct, real-time measurement of rapid inorganic phosphate release using a novel fluorescent probe and its application to actomyosin subfragment 1 ATPase. Biochemistry.

[CR13] Bürker K, Tigerstedt R (1908). Methoden zur Thermodynamik des Muskels. Handbuch der physiologischen Methodik. S.

[CR14] Bürker K (1919). Experimentelle Untersuchungen zur Thermodynamik des Muskels. Pflügers Arch.

[CR15] Cain DF, Davies RE (1962). Breakdown of adenosine triphosphate during a single contraction of working muscle. Biochem Biophys Res Commun.

[CR16] Cain DF, Infante AA, Davies RE (1962). Chemistry of muscle contraction. Adenosine triphosphate and phosphorylcreatine as energy supplies for single contractions of working muscle. Nature.

[CR17] Caremani M, Melli L, Dolfi M, Lombardi V, Linari M (2015). Force and number of myosin motors during muscle shortening and the coupling with the release of the ATP hydrolysis products. J Physiol.

[CR18] Curtin NA, Woledge RC (1979). Chemical change and energy production during contraction of frog muscle: how are their time courses related?. J Physiol.

[CR19] Curtin NA, Woledge RC (1991). Efficiency of energy conversion during shortening of muscle fibres from the dogfish *Scyliorhinus canicula*. J Exp Biol.

[CR20] Curtin NA, Gilbert C, Kretzschmar KM, Wilkie DR (1974). The effect of the performance of work on total energy output and metabolism during muscular contraction. J Physiol.

[CR21] Ebashi S, Endo M (1968). Calcium ion and muscle contraction. Prog Biophys Mol Biol.

[CR22] Evans CL, Hill AV (1914). The relation of length to tension development and heat production on contraction in muscle. J Physiol.

[CR23] Fenn WO (1923). A quantitative comparison between the energy liberated and the work performed by isolated sartorius muscle of the frog. J Physiol.

[CR24] Fenn WO (1924). The relation between the work performed and the energy liberated in muscular contraction. J Physiol.

[CR25] Fenn WO, Marsh BS (1935). Muscular force at different speeds of shortening. J Physiol.

[CR26] Ferenczi MA (1986). Phosphate burst in permeable muscle fibers of the rabbit. Biophys J.

[CR27] Ferenczi MA, Homsher E, Trentham DR (1984). The kinetics of magnesium adenosine triphosphate cleavage in skinned muscle fibres of the rabbit. J Physiol.

[CR28] Ferenczi MA, He ZH, Chillingworth RK, Brune M, Corrie JE, Trentham DR, Webb MR (1995). A new method for the time-resolved measurement of phosphate release in permeabilized muscle fibers. Biophys J.

[CR29] Fick A (1881). The development of heat by muscular activity. Science.

[CR30] Fick A (1892). Neue Beiträge zur Kenntniss von der Wärmeentwickelung im Muskel. Pflügers Arch.

[CR31] Fiske CH, Subbarow Y (1929). Phosphorus compounds of muscle and liver. Science.

[CR32] Ford LE, Huxley AF, Simmons RM (1985). Tension transients during steady shortening of frog muscle fibres. J Physiol.

[CR33] Gasser HS, Hill AV (1924). The dynamics of muscular contraction. Proc R Soc Lond B.

[CR34] Glyn H, Sleep J (1985). Dependence of adenosine triphosphatase activity of rabbit psoas muscle fibres and myofibrils on substrate concentration. J Physiol.

[CR35] Hartree W (1924). The measurement of small rates of heat production by thermopile and galvanometer. J Sci Instrum.

[CR36] Hartree W (1925). An analysis of the heat production during a contraction in which work is performed. J Physiol.

[CR37] Hartree W, Hill AV (1928). The energy liberated by an isolated muscle during the performance of work. Proc R Soc Lond B.

[CR38] Hartree W, Hill AV (1928). The factors determining the maximum work and the mechanical efficiency in muscle. Proc R Soc Lond B.

[CR39] He ZH, Chillingworth RK, Brune M, Corrie JE, Trentham DR, Webb MR, Ferenczi MA (1997). ATPase kinetics on activation of rabbit and frog permeabilized isometric muscle fibres: a real time phosphate assay. J Physiol.

[CR40] He ZH, Chillingworth RK, Brune M, Corrie JE, Webb MR, Ferenczi MA (1999). The efficiency of contraction in rabbit skeletal muscle fibres, determined from the rate of release of inorganic phosphate. J Physiol.

[CR41] He ZH, Bottinelli R, Pellegrino MA, Ferenczi MA, Reggiani C (2000). ATP consumption and efficiency of human single muscle fibers with different myosin isoform composition. Biophys J.

[CR42] Heidenhain R (1869). Ueber Ad. Fick's experimentellen Beweis für die Gültigkeit des Gesetzes von der Erhaltung der Kraft bei der Muskelzusammenziehung. Pflügers Arch.

[CR43] Helmholtz H (1848) Ueber die warmeentwickelung bei der muskelaction. Archiv für Anatomie, Physiologie und Wissenschaftliche Medicin 144–164

[CR44] Hill AV (1910). The heat produced in contracture and muscular tone. J Physiol.

[CR45] Hill AV (1911). The position occupied by the production of heat, in the chain of processes consituting a muscular contraction. J Physiol.

[CR46] Hill AV (1913). The absolute mechanical efficiency of the contraction of an isolated muscle. J Physiol.

[CR47] Hill AV (1913). The energy degraded in the recovery processes of stimulated muscles. J Physiol.

[CR48] Hill AV (1920). An instrument for recording the maximum work in a muscular contraction. J Physiol.

[CR49] Hill AV (1922). The maximum work and mechanical efficiency of human muscles, and their most economical speed. J Physiol.

[CR50] Hill AV (1925). Length of muscle, and the heat and tension developed in an isometric contraction. J Physiol.

[CR51] Hill AV (1928). The diffusion of oxygen and lactic acid through tissue. Proc R Soc Lond B.

[CR52] Hill AV (1937). Methods of analysing the heat production of Muscle. Proc R Soc Lond B.

[CR53] Hill AV (1938). Energy liberation and “viscosity” in muscle. J Physiol.

[CR54] Hill AV (1938). Heat of shortening and the dynamic constants of muscle. Proc R Soc Lond B.

[CR55] Hill AV (1939). The transformations of energy and the mechanical work of muscles. Proc Phys Soc.

[CR56] Hill AV (1949). Myothermic methods. Proc R Soc Lond B.

[CR57] Hill AV (1949). Work and heat in a muscle twitch. Proc R Soc Lond B.

[CR58] Hill AV (1953). A reinvestigation of two critical points in the energetics of muscular contraction. Proc R Soc Lond B.

[CR59] Hill AV (1959). The heat production of muscle and nerve, 1848–1914. Ann Rev Physiol.

[CR60] Hill AV (1964). The effect of load on the heat of shortening of muscle. Proc R Soc Lond B.

[CR61] Hill AV (1965). The mechanism of muscle contraction. Nobel lectures, physiology or medicine.

[CR62] Hill AV (1965). Trails and trials in physiology.

[CR63] Hill AV, Hartree W (1920). The four phases of heat-production of muscle. J Physiol.

[CR64] Hill AV, Woledge RC (1962). An examination of absolute values in myothermic measurements. J Physiol.

[CR65] Homsher E (1987). Muscle enthalpy production and its relationship to actomyosin ATPase. Annu Rev Physiol.

[CR66] Homsher E, Mommaerts WF, Ricchiuti NV, Wallner A (1972). Activation heat, activation metabolism and tension-related heat in frog semitendinosus muscles. J Physiol.

[CR67] Homsher E, Kean CJ, Wallner A, Garibian-Sarian V (1979). The time-course of energy balance in an isometric tetanus. J Gen Physiol.

[CR68] Homsher E, Yamada T, Wallner A, Tsai J (1984). Energy balance studies in frog skeletal muscles shortening at one-half maximal velocity. J Gen Physiol.

[CR69] Homsher E, Lacktis J, Regnier M (1997). Strain-dependent modulation of phosphate transients in rabbit skeletal muscle fibers. Biophys J.

[CR70] Huxley AF (1957). Muscle structure and theories of contraction. Prog Biophys Biophys Chem.

[CR71] Huxley H, Hanson J (1954). Changes in the cross-striations of muscle during contraction and stretch and their structural interpretation. Nature.

[CR72] Jensen WB (2010). Why are q and Q used to symbolize heat?. J Chem Educ.

[CR73] Katz B (1978). Archibald Vivian Hill. Biogr Mems Fell R Soc.

[CR74] Kushmerick MJ, Peachey LE (1983). Energetics of muscle contraction. Handbook of physiology: skeletal muscle.

[CR75] Kushmerick MJ, Davies RE (1969). The chemical energetics of muscle contraction. II. The chemistry, efficiency and power of maximally working sartorius muscles. Appendix free energy and enthalpy of ATP hydrolysis in the sarcoplasm. Proc R Soc Lond B.

[CR76] Levin AM, Wyman J (1927). The viscous elastic properties of muscle. Proc R Soc Lond B.

[CR77] Liebig J (1842). Animal chemistry or chemistry in its applications to physiology and pathology.

[CR78] Linari M, Caremani M, Piperio C, Brandt P, Lombardi V (2007). Stiffness and fraction of myosin motors responsible for active force in permeabilized muscle fibers from rabbit psoas. Biophys J.

[CR79] Linari M (2015). Force generation by skeletal muscle is controlled by mechanosensing in myosin filaments. Nature.

[CR80] Lohmann K (1929). Uber die pyrophosphatfraktion im muskel. Naturwissenschaften.

[CR81] Mommaerts WFHM (1969). Energetics of muscular contraction. Physiol Rev.

[CR82] Mommaerts WFHM (1970). What is the Fenn effect?. Naturwissenschaften.

[CR83] Nawalichin J (1877). Myothermische Untersuchungen. Pflügers Arch.

[CR84] Needham DM (1950). Myosin and adenosine triphosphate in relation to muscle contraction. Biochem Biophys Acta.

[CR85] Needham DM (1971). Machina Carnis. The biochemistry of muscular contraction in its historical development.

[CR86] Offer G, Ranatunga KW (2010). Crossbridge and filament compliance in muscle: implications for tension generation and lever arm swing. J Muscle Res Cell Motil.

[CR87] Potma EJ, Stienen GJ (1996). Increase in ATP consumption during shortening in skinned fibres from rabbit psoas muscle: effects of inorganic phosphate. J Physiol.

[CR88] Rall JA (1982). Sense and nonsense about the Fenn effect. Am J Physiol Heart Circ Physiol.

[CR89] Rall JA (2017). Nobel Laureate A. V. Hill and the refugee scholars, 1933–1945. Adv Physiol Educ.

[CR90] Rall JA (2018). Mechanism of muscle contraction. Perspectives in physiology.

[CR91] Reggiani C, Potma EJ, Bottinelli R, Canepari M, Pellegrino MA, Stienen GJM (1997). Chemo-mechanical energy transduction in relation to myosin isoform composition in skeletal muscle fibres of the rat. J Physiol.

[CR92] Smith ICH (1972). Energetics of activation in frog and toad muscle. J Physiol.

[CR93] Sun YB, Hilber K, Irving M (2001). Effect of active shortening on the rate of ATP utilisation by rabbit psoas muscle fibres. J Physiol.

[CR94] Tsianos GA, MacFadden LN (2016). Validated predictions of metabolic energy consumption for submaximal effort movement. PLoS Comput Biol.

[CR95] Tsianos GA, Rustin C, Loeb GE (2012) Mammalian muscle model for predicting force and energetics during physiological behaviors IEEE transactions on neural systems and rehabilitation engineering : a publication of the IEEE Engineering in Medicine and Biology Society 20:117-133. 10.1109/TNSRE.2011.2162851 10.1109/TNSRE.2011.2162851 21859633

[CR96] Umberger BR, Gerritsen KG, Martin PE (2003). A model of human muscle energy expenditure. Comput Methods Biomech Biomed Engin.

[CR97] West TG, Curtin NA, Ferenczi MA, He ZH, Sun YB, Irving M, Woledge RC (2004). Actomyosin energy turnover declines while force remains constant during isometric muscle contraction. J Physiol.

[CR98] Wilson WH, Epps TD (1919). The construction of thermo-couples by electro-deposition. Proc Phys Soc London.

[CR99] Winters JA, Winters JM, Woo SL-Y (1990). Hill-based muscle models: A systems engineering perspective. Multiple muscle systems. Biomechanics and movement organisation.

[CR100] Woledge RC (1968). The energetics of tortoise muscle. J Physiol.

[CR101] Woledge RC, Curtin NA, Homsher E (1985) Energetic aspects of muscle contraction vol 41. Monographs of the Physiological Society. Academic Press, London 3843415

[CR102] Wyman J (1926). Studies on the relation of work and heat in tortoise muscle. J Physiol.

